# How to Help Us

**Published:** 1888-12

**Authors:** 


HOW TO HELP US: Those of our subscribers who are in arrears with their subscriptions, could aid us very much by prompt remittance. Do as you would be done by!

